# A method to preserve low parasitaemia *Plasmodium*-infected avian blood for host and vector infectivity assays

**DOI:** 10.1186/s12936-016-1198-5

**Published:** 2016-03-11

**Authors:** Jenny S. Carlson, Federico Giannitti, Gediminas Valkiūnas, Lisa A. Tell, Joy Snipes, Stan Wright, Anthony J. Cornel

**Affiliations:** Mosquito Control Research Laboratory, Department of Entomology and Nematology, Kearney Agriculture Center, University of California, Parlier, Davis, USA; Veterinary Diagnostic Laboratory, Veterinary Population Medicine Department, College of Veterinary Medicine, University of Minnesota, Saint Paul, USA; Instituto Nacional de Investigación Agropecuaria, La Estanzuela, Colonia, Uruguay; California Animal Health and Food Safety Laboratory, School of Veterinary Medicine, University of California, Davis, USA; Nature Research Centre, Akademijos 2, 08412 Vilnius, Lithuania; Department of Medicine and Epidemiology, School of Veterinary Medicine, University of California, Davis, USA; Sacramento-Yolo Mosquito Vector and Control District, Elk Grove, USA; Vector Genetics Laboratory, Department of Pathology, Microbiology and Immunology, University of California, Davis, USA

**Keywords:** Avian malaria, Experimental infection, *Plasmodium cathemerium*, Blood preservation, Bird inoculation, Pathology, *Culex* spp. vectors

## Abstract

**Background:**

Avian malaria vector competence studies are needed to understand more succinctly complex avian parasite-vector-relations. The lack of vector competence trials may be attributed to the difficulty of obtaining gametocytes for the majority of *Plasmodium* species and lineages. To conduct avian malaria infectivity assays for those *Plasmodium* spp. and lineages that are refractory to in vitro cultivation, it is necessary to obtain and preserve for short periods sufficient viable merozoites to infect naïve donor birds to be used as gametocyte donors to infect mosquitoes. Currently, there is only one described method for long-term storage of *Plasmodium* spp.—infected wild avian blood and it is reliable at a parasitaemia of at least 1 %. However, most naturally infected wild-caught birds have a parasitaemia of much less that 1 %. To address this problem, a method for short-term storage of infected wild avian blood with low parasitaemia (even ≤0.0005 %) has been explored and validated.

**Methods:**

To obtain viable infective merozoites, blood was collected from wild birds using a syringe containing the anticoagulant and the red blood cell preservative citrate phosphate dextrose adenine solution (CPDA). Each blood sample was stored at 4 °C for up to 48 h providing sufficient time to determine the species and parasitaemia of *Plasmodium* spp. in the blood by morphological examination before injecting into donor canaries. *Plasmodium* spp.—infected blood was inoculated intravenously into canaries and once infection was established, *Culex stigmatosoma*, *Cx. pipiens* and *Cx. quinquefasciatus* mosquitoes were then allowed to feed on the infected canaries to validate the efficacy of this method for mosquito vector competence assays.

**Results:**

Storage of *Plasmodium* spp.—infected donor blood at 4 °C yielded viable parasites for 48 h. All five experimentally-infected canaries developed clinical signs and were infectious. Pathologic examination of three canaries that later died revealed splenic lesions typical of avian malaria infection. Mosquito infectivity assays demonstrated that *Cx. stigmatosoma* and *Cx. pipiens* were competent vectors for *Plasmodium cathemerium.*

**Conclusions:**

A simple method of collecting and preserving avian whole blood with malaria parasites of low parasitaemia (≤0.0005 %) was developed that remained viable for further experimental bird and mosquito infectivity assays. This method allows researchers interested in conducting infectivity assays on target *Plasmodium* spp. to collect these parasites directly from nature with minimal impact on wild birds.

## Background

Studies in avian malaria traditionally have been heavily biased towards investigating and understanding avian host-parasite relationships. Studies on vector-parasite relationships, in contrast, have been less investigated because there is a lack of available colonies of many vector mosquito species and secondly, because cultures of most avian *Plasmodium* species and lineages are not available. However, the determination of whether or not a particular vector is a competent vector is an important factor when considering the epizootiology of avian malaria.

Vector and host competence assays require a source of infective blood, which cannot easily be done for most of the approximately 50 avian *Plasmodium* spp. that have been described to date [1, 2]. Most avian *Plasmodium* species and lineages cannot be established in vitro and are refractory to or difficult to cultivate [3]. Currently, there is only a hand full of *Plasmodium* spp. that can be cultured and maintained in vitro and they are *Plasmodium lophurae* [3–5], *P. hexamerium* [6], *P. gallinaceum* [7–10], *P. relictum* [11–13], *P. elongatum* [13] and *P. circumflexum* [14]. To conduct avian malaria infectivity assays on *Plasmodium* spp. for which there are no cultures, parasites must be obtained directly from nature and be viably transported and preserved until the experimental infectivity assays can be carried out.

Vector competence is an important factor in overall vectorial capacity [15, 16], which broadly defines the efficiency of mosquito species to transmit arthropod borne pathogens. Currently, separation of major and minor avian malaria vectors are mainly based on either whole body or midgut infection prevalence rates in field-caught mosquitoes. Reliance on prevalence rates unfortunately, has inherent issues that can lead to misleading estimates of vector competence. Abortive haemosporidian infections commonly occur, and PCR-based tools do not target identification of viable sporozoites specifically and may only recognize gut oocyst limited infections [2]. Furthermore, sampling can skew results due to small sample sizes, pooling of samples, reliance on *Plasmodium* detection in midguts and not salivary gland infections, crude identification of the mosquito hosts (especially for species complexes), and variable methods used to capture the mosquitoes of different physiological states and age.

It became recently apparent, from mosquito prevalence data, that several laboratory-based infectivity studies were needed to learn more about potential vector incompetency phenomena occurring in avian Californian *Plasmodium* and certain *Culex* mosquito species [17]. These apparent vector incompatibility issues may be real or due to misleading interpretations derived from inherent issues of sampling as mentioned above, which can be resolved by laboratory vector competence trials. Vector incompatibilities were noted to potentially occur in *Plasmodium* species that infect birds at a low parasitaemia and for which there are no cultures. The current cryopreservation of infected blood derived from birds usually is effective only if the parasitaemia is greater than 1 % [18], a parasitaemic level rarely seen in any of the *Plasmodium* species of interest in birds in the study area. This required developing a method to isolate and maintain low parasite levels of *Plasmodium* from wild birds for later viable mosquito vector competence trials.

Currently, there are two major ways in which avian malaria parasites can be obtained from birds found in nature. The first method is to place sentinel birds at a location where local transmission of targeted parasites has been documented. Sentinel birds require a cage that protects them from potential predators, but that will simultaneously allow mosquitoes to successfully enter. Weekly samples from these birds are necessary during the months when transmission takes place. Screening for parasites from birds requires obtaining a blood sample, making and staining a thin blood smear with Giemsa, and then identifying parasites morphologically by microscopy [19] or by PCR and sequencing. If sentinel birds are used and test positive for infection, they can either be brought back to the laboratory and its blood used as a donor of infected blood to naïve birds, or they could be used to directly provide an infectious blood meal to mosquitoes if mosquito species of interest are readily available. However, this presents several difficulties. The sentinel birds require daily care in accordance with the Institutional Animal Care and Use Committee (IACUC) protocols, which can be time-consuming and logistically difficult to carry out. If a sentinel location has local transmission of multiple *Plasmodium* spp. and other avian pathogens, then there is a high risk that the sentinel birds will be exposed to multiple co-infections. Due to specificity and limited host range of many *Plasmodium* spp. a common sentinel bird species (canaries, ducklings) often cannot be used, because they may not attract all vector species and may be resistant to many *Plasmodium* species [20]. The use of wild birds as sentinels is often difficult, because of permit restrictions and difficulties of maintaining and transporting them in captivity.

The second method by which parasites can be obtained from nature is to use wild birds directly from a field site where the target *Plasmodium* spp. has been reported. Wild birds can be tested for infection in the same way as described for sentinel birds. There are two ways in which a wild bird could be used as a source of infected blood. If IACUC approval has been granted for the holding, the transportation and laboratory maintenance of a wild bird, then the bird can be held for the duration of the staining process of the blood and determination of infection status. Once an infected bird has been identified, it can be used as a donor of infected blood to a susceptible species. However, if IACUC approval has not been granted for such holding and transportation, which is often the case, then the blood collected from the wild birds can be refrigerated and preserved for a short period (several days) basis, or for a long term (many years) by freezing in liquid nitrogen [18, 21, 22]. However, there is no current protocol described for the short-term preservation of infected blood by refrigeration. The difficulties with using wild birds lies with the probability of finding an individual that is infected with the target *Plasmodium* spp. especially if it is a species that occurs at a low prevalence and parasitaemia in the bird population.

In an attempt to attain avian *Plasmodium* spp. from the field in California to perform malaria infectivity assays in a multitude of mosquito species, a method was developed for short-term preservation of avian whole blood with low *Plasmodium* parasitaemia for direct infection of experimental birds. Pathologic changes in experimentally exposed canaries are described. Moreover, an experimental infection of three mosquitoes species, *Cx. stigmatosoma, Cx. pipiens* and *Cx. quinquefasciatus* is reported confirming that inoculation of canaries with the described method resulted in a subsequent gametocytaemia and successful mosquito infection.

## Methods

### Bird trapping, blood collection and transport

Blood collections from birds were conducted regularly from February to September of 2014 at the Stone Lakes National Wildlife Refuge (NWR). Stone Lakes NWR is a basing floodplain that supports thousands of resident and migratory birds. Infected blood was obtained from three locations at Stone Lakes NWR: Keyhole (38°25′40.9″N, 121°29′57.2″W), Lewis pond (38°25′24.9″N 121°29′22.7″W) and Peninsula central loop (38°20′38.1″N, 121°30′14.1″W). Birds were trapped using mist nets. Upon capture, each bird was identified to species level, measured, weighed and banded. A blood sample of approximately 100 μl or ≤1 % of the body weight was obtained from each bird by jugular venipuncture with a syringe that already contained one of two different types of anticoagulant tested—heparin or citrate phosphate dextrose adenine (CPDA) solution. An aliquot of blood was used to make two to three thin blood smears that were air-dried and fixed in absolute methanol in the field, and brought back to the laboratory. The remainder of the blood was split, with half immediately stored in lysis buffer (10 mM Tris-HCL, pH 8.0, 100 mM EDTA, 2 % SDS) at room temperature for later DNA extraction and molecular testing, and half left in the syringe and stored in a plastic bag on top of wet ice, with a few paper towels separating the ice and the bagged syringes. All slides were brought back to the laboratory and were stained the same day they were collected with Giemsa as described by Valkiūnas et al. [19]. A 48-hour turnaround period was imposed from the time the blood samples were collected to the time they could be used for inoculation into naïve canaries. Within this time frame the slides were stained, viewed, and if deemed positive, the intensity of parasitaemia estimated by counting of the number of parasites per 1000 erythrocytes or per 10,000 erythrocytes when parasitaemia was low (<0.1 %).

A wide range of blood anticoagulants with different modes of action are commercially available. As stated by Vaught [23], it is important to carefully choose the appropriate anticoagulant for the work at hand, especially to circumvent complications with blood that has been transported from the field and inoculated into a naïve host. In this study, two different anticoagulant solutions were tested: heparin solution and CPDA. The IACUC committee approved the use of 40 canaries for this and vector competence studies (UC Davis IACUC protocol 17601), but only 10 were obtained from a breeder for this experiment, which limited evaluating numerous anticoagulants and red blood cell preservatives. Heparin inactivates thrombin and other clotting factors by binding to antithrombin III [23]. CPDA contains citric acid, sodium citrate, monobasic sodium phosphate, dextrose, and adenine, and it improves red blood cell preservation and whole blood storage up to 35 days by providing adenine, needed for the maintenance of adenosine triphosphate (ATP), which prolongs red blood cell survival [24].

Heparin was used initially, but the first inoculation attempt into a canary, from blood collected from the Peninsula Central loop site on March 12th 2014 from a Puget Sound White-crowned Sparrow (PSWS; *Zonotrichia leucophrys pugetensis*), almost instantaneously killed the canary (canary 0503). The remaining blood from this PSWS sample was used to make thin blood smears, which when examined was found to have considerable clumping of thrombocytes, which likely was the reason why the canary died. CPDA was used instead of heparin, because CPDA increases the survival time of red blood cells [24]. All needles were initially prepped with 0.014 cc of CPDA, which was kept in a portable transport cooler (styrofoam box containing a bag of ice) before taking a blood sample from each bird. Upon arrival at the laboratory, all syringes were were immediately stored in a refrigerator at 4 °C.

After drawing blood from the jugular vein, the whole blood was stored in the syringe during transport, and was not expelled into a tube. During preliminary attempts to transport blood, it was determined that when blood was ejected into both spray-coated sodium heparin (green cap) and spray-coated K_2_EDTA (lavender cap) plastic tubes (BD, Franklin Lakes, NJ), there was an increased chance of thrombocyte clumping.

### Interspecies blood compatibility

As stated by Shimmel et al. [25], inoculating blood into a recipient of a different species (heterologous blood transfusion) carries some degree of health risk or even death, due to blood type incompatibilities. There are different blood types in all animal species [25], but birds may have the most complex blood groupings [26, 27]. Therefore, it is then prudent to first conduct blood compatibility assays between donor and recipient birds. Due to the death of canary 0503 mentioned above, it was decided to also eliminate the possibility that blood incompatibility was the cause of death. Thus, compatibility between donor White-crowned sparrow (WCSP, *Zonotrichia leucophrys*), Black-headed grosbeaks (BHGR, *Pheucticus melanocephalus*) and Fox sparrow (FOSP, *Passerella iliaca*) and the recipient canary (*Serinus canaria domestica*) blood was determined by an agglutination crossmatching assay following the protocols described by Lichtenberger [28] and Martinho [29]. There was no haemagglutination of the red blood cells in the minor or the major crossmatching, indicating that the blood types were compatible.

### Determination of infection

Parasites were identified both by microscopy within the first 48 h after collection using morphological keys in Valkiūnas [4], and later by sequencing the parasite’s DNA. The criteria for selecting wild bird blood for inoculation into canaries included: A—a minimum visualization of two meronts per 10,000 erythrocytes (parasitaemia = 0.0002 %), and B—identification of the gametocytes to species level.

To molecularly confirm identification of *Plasmodium* species within blood samples after they were injected into canaries, parasite DNA was amplified by PCR for sequencing purposes. Parasite DNA was extracted from avian whole blood samples following the DNeasy blood and tissue kit protocols (Qiagen, Valencia, California). A nested PCR described in Waldenström et al. [30] was carried out to amplify a 478 bp sequence of the mitochondrial cytochrome oxidase subunit-*b* gene (cyt *b*). PCRs were carried out in a 20 μl reaction mixture using AccuPower Taq PCR PreMix (Bioneer Corporation, Daejeon, Republic of Korea) containing 1 μl of each 10 μM primer, 0.5 μl of Bovine Serum Albumin (BSA), 2 μl of template DNA and 15.5 μl of purified water. All PCR products were viewed on 1.8 % agarose gels stained with ethidium bromide. Positive samples were cleaned and sent for sequencing to Elim Biopharmaceuticals Inc. (Hayward, California) and sequences were edited using Sequencher 5.1 (GeneCodes, Ann Arbor, Michigan). Consensus sequences then were identified using the NCBI Nucleotide Blast search.

### Inoculation of donor blood into canaries

Ten domestic canaries were purchased from a commercial breeder and kept in a mosquito free room (quarantine) for one month at the Center for Vectorborne Diseases Biosafety Level 3 Laboratory (UC Davis). Canaries were tested for any pre-existing avian malaria infections upon arrival at the laboratory, 15 and 30 days after arrival. Microscopic examinations and PCR screening (methods described previously in the determination of infection section) revealed that all 10 canaries were not infected with malaria parasites prior to the experimental inoculation.

**Fig. 1:**
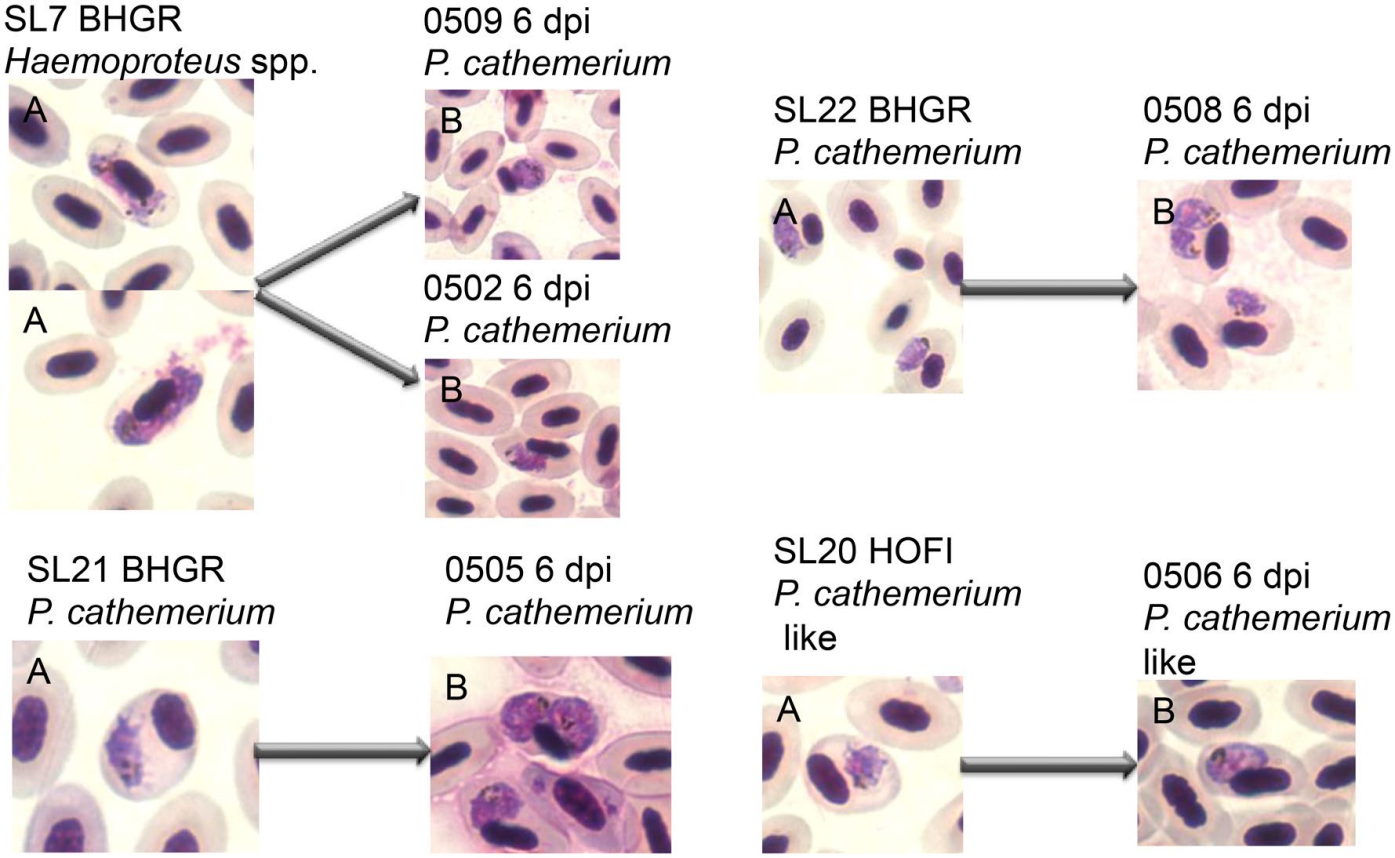
Parasite identification and bird blood inoculation scheme. The pictures are of parasites found in Giemsa-stained blood smears. Parasite pictures labelled *A* represent the donor blood, where SL stands for Stone Lakes (the location where the bird was collected) and the ID of the bird, BHGR stands for Black-headed Grosbeak (*Pheucticus melanocephalus*) and HOFI stands for House Finch (*Haemorhous mexicanus*). Pictures labelled *B* represent the blood taken six days post infection (dpi) from the experimental canaries. It is important to note that sample SL7 (BHGR) was originally infected with *Haemoproteus* spp., but *P. cathemerium* was not visualized during microscopic screening. Canaries 0502 and 0509 did not become infected with *Haemoproteus* as expected, but *P. cathemerium* successfully established an infection

A total of 203 wild bird blood samples were collected, four of which were selected for inoculation once the switch from heparin to the anticoagulant CPDA was made—samples SL7 BHGR, SL21 BHGR, SL22 BHGR, and SL20 HOFI (House Finch, *Haemorhous mexicanus*) (Fig. 1). Blood sample SL7 BHGR was used to evaluate if blood stored for 48 h post-extraction and preserved with CDPA had any ill effects in a recipient canary. This sample had a few red blood cells infected with a *Haemoproteus* spp. which would not be expected to elicit any ill effects in canaries because *Haemoproteus* gametocytes do not multiply and are non-infective unlike *Plasmodium* meronts, which produce infective merozoites. After no overt ill effects of CPDA were observed with blood sample SL7, three other canaries were infected with the remaining three CPDA blood samples that were found microscopically to be infected with *P. cathemerium* and *P. cathemerium*-like parasites (Fig. 1). All four inoculations into recipient canaries were carried out using 0.014 cc of CPDA mixed with blood and were injected intravenously into the jugular vein. A blood sample was obtained from canaries on days three and five post-inoculation to determine the status of infection by microscopic examination of Giemsa-stained blood smears.

Sample SL7 was collected from the Keyhole site on April 23rd 2014 from a BHGR and it was used to inoculate two canaries, 0502 and 0509, on April 24th. The estimated parasitaemia was 0.0002 % and the parasite was identified as a *Haemoproteus* spp. No microscopic or PCR-based evidence indicated that a parasite of the genus *Plasmodium* was present in this sample. As expected, *Haemoproteus* did not establish an infection in the two canaries. However, despite the inability to visually or genetically detect *Plasmodium* parasites on initial screening, *P. cathemerium* trophozoites were visible in red blood cells five days post infection (dpi) from both canaries. The parasite was identified as *P. cathemerium*, because samples from the canary infection matched lineage SPTO_CA_ELW_6P [Genbank, KJ620779] reported by Carlson et al. [17] and Walther et al. [31], and had a 100 % match to Genbank submission AY377128 of the cyt *b* gene for *P. cathemerium* by Wiersch et al. [32].

Samples SL21 and SL22 were both collected from the Peninsula central loop on June 12th 2014 from two BHGRs, and were used to inoculate canaries 0505 and canary 0508 respectively on June 13th. The estimated parasitaemia for SL21 and SL22 were 0.0005 and 0.0024 % respectively. The parasite was also identified genetically as *P. cathemerium* (lineage SPTO_CA_ELW_6P).

Sample SL20 was collected from the Lewis trail site on July 2nd 2014 from a HOFI, and was used to inoculate canary 0506 on July 3rd. The estimated parasitaemia was 0.0005 % and this lineage, named HOFI_CA_JSC_1P [Genbank, KT067675], was identified as *P. cathemerium*-like. The genetic distance between this lineage and the SPTO_CA_ELW_6P lineage was 1.05 %, but these parasites were indistinguishable morphologically.

### Phylogenetic analysis of *Plasmodium* parasites

**Fig. 2:**
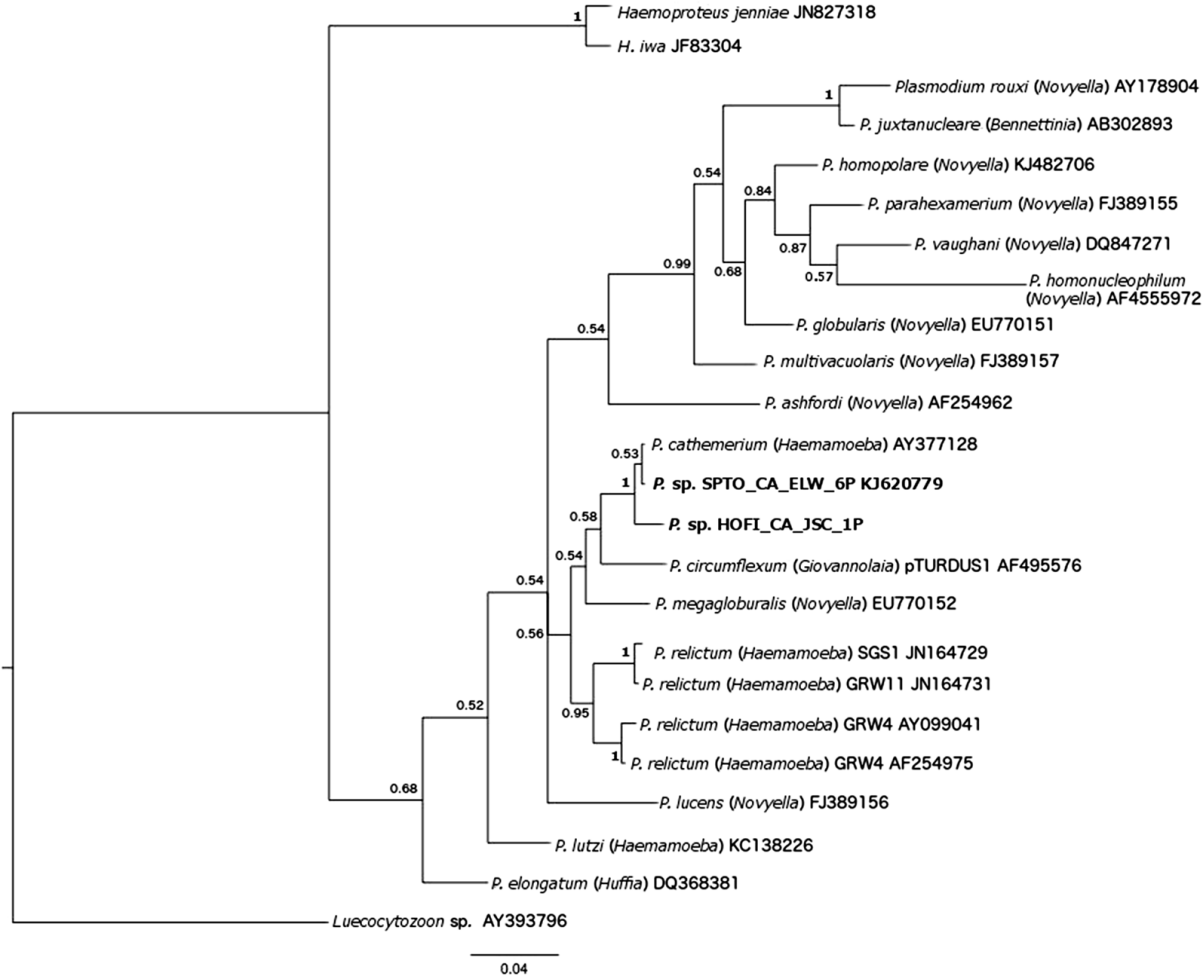
Bayesian phylogeny of 21 mitochondrial cytochrome *b* *Plasmodium* spp./lineages and two *Haemoproteus* spp. The *Leucocytozoon* sp. lineage was used as the out-group. Numbers at each node represent the Bayesian posterior probabilities. Lineage names used in experiments are given in bold; they were obtained from birds collected at the Stone Lakes National Wildlife Refuge. All *Plasmodium* species/lineages used for this analysis are delineated by the parasite name with the subgenus in parenthesis and followed by the Genbank accession number

Parasite sequences obtained in this study were added to a dataset containing 19 other cyt *b Plasmodium* spp. sequences obtained from GenBank (Fig. 2). The software MrModeltest [33] was used to determine the best fit model of sequence evolution of GTR + Γ. MrBayes version 3.1.2 [34] was used to construct a Bayesian phylogeny. A total of 10 million generations were ran with a sample frequency of every 200 generations. During the construction of the majority consensus tree, 25 % of the initial trees were discarded and the remaining trees were used to construct the majority rule consensus tree and to calculate the posterior probabilities of the individual clades.

### Collection and rearing of field mosquitoes

Adult female *Cx. pipiens, Cx. quinquefasciatus* and *Cx. stigmatosoma* mosquitoes used in the experimental infection were collected in gravid traps at China Creek Park in Fresno County, California (36°43′27.0″N, 119°30′07.3″W). Gravid traps were infused with 7-day old grass as an attractant [35] and species were identified morphologically using dichotomous keys in Darise and Ward [36] and in Bohart and Washino [37]. All gravid females were brought back to the laboratory where they were provided with a cup of water containing vegetation to lay their eggs in. Egg rafts were hatched and reared from larvae to adults at the Mosquito Control Research Laboratory.

### Experimental infection of mosquitoes

Reared adult mosquitoes were transported to the Center for Vectorborne Diseases Biosafety Level 3 Laboratory. All mosquitoes were held in 3.8 L plastic buckets with tops covered in fine mesh and held at 26 °C and ca. 70 % humidity with a 12 h light/dark cycle in an incubator. Up to 40 mosquitoes were placed in each bucket and were provided with cotton balls lightly saturated in 10 % sucrose on top of the mesh as a source of sustenance.

To increase the probability of blood-feeding, mosquitoes were starved for 24 h by removing the 10 % sucrose cotton balls. *Cx. stigmatosoma* and *Cx. pipiens* complex were kept separate throughout the entire feeding process to avoid interspecific competition to the host. Up to 100 mosquitoes of one species were transferred from their original cage to the experimental 3.7 L bucket that had an infected canary in it, and then it was repeated for the second mosquito species. Experimental infection buckets had, in addition to the meshed top, a cloth sleeved opening large enough to insert a canary. The canaries were left unrestrained in the experimental bucket, and once the mosquitoes were added, the sleeve was tied with a knot and the laboratory lights were dimmed. The mosquitoes were allowed to feed for an hour on each canary starting at 20:00 hours to emulate their nocturnal feeding pattern. The feeding was supervised for the entire hour to ensure that no more than 50 mosquitoes would feed on one canary at a time, to follow IACUC restrictions.

Fully blooded mosquitoes were transferred into smaller half-liter cartons (≤25 individuals per carton) and returned to the incubator. Non-blood-fed mosquitoes were placed back into their original one-gallon buckets, to be used in subsequent further feeding attempts. Partially blood-fed mosquitoes were discarded. All mosquitoes were monitored daily, and any individuals that died prior to a scheduled post infection time point for dissection were removed and placed into 70 % ethanol until processed with the experimental mosquito samples.

### Determination of infection in experimental mosquitoes

To determine a time series infectious status of all mosquitoes, a subset of each group were dissected on 10, 16, 20, 25, 26, and/or 30 dpi. Midgut and salivary gland dissections were made from triethylamine (Sigma-Aldrich, St. Louis, MO) anesthetized mosquitoes. The remaining thorax from each mosquito was preserved separately in tubes filled with 70 % ethanol for later DNA extraction and parasite DNA amplification in the same manner described by Carlson et al. [17]. Salivary gland slide preparations were made and fixed according to Kazlauskienė et al. [38]. Midguts were pulled out of abdomens and placed in a drop of saline on the glass slide, followed by a drop of 0.5 % solution of mercurochrome and then five min later viewed under a microscope to count oocysts. DNA extracted from *Cx. pipiens* complex mosquito thoraxes was used to distinguish between *Cx. pipiens*, *Cx. quinquefasciatus*, and hybrids of the two species by PCR following the protocol described by Smith et al. [39]. Chi square test for independent samples was performed in RStudio statistical software package, version 0.98.1091 (RStudio Foundation for Statistical Computing; Vienna, Austria) to determine statistical significance of infection status between *Culex* species (P ≤ 0.05 was considered significant).

### Pathologic examination of deceased birds

To assess the pathogenicity of the *P. cathemerium* strains under investigation, pathologic examinations were performed on three experimentally-inoculated canaries that showed clinical signs and succumbed to the disease at 21, 16, and 30 dpi (canaries 0502, 0509, and 0505 respectively, all infected with *P. cathemerium* lineage SPTO_CA_ELW_6P), and one age-matched uninfected control that died of natural causes (canary 0507). Clinical signs in the inoculated birds included having puffed up feathers, reluctance to eat and move, and impaired balance. The whole carcasses were immersion-fixed in one l of 10 % neutral buffered formalin solution for 48 h and submitted to the California Animal Health and Food Safety (CAHFS) laboratory of the University of California, Davis for postmortem examination. At necropsy, major organs were examined and the length and diameter of the spleen were measured. Samples of all major organs were collected and processed routinely, embedded in paraffin, sectioned at four microns, mounted on glass slides, and stained with haematoxylin and eosin for histologic (microscopic) examination, following CAHFS standard operating procedures. Additionally, formalin-fixed paraffin-embedded tissue sections were processed for the detection of *Sarcocystis neurona*, *Sarcocystis falcatula*, and *Chlamydia* spp. by immunohistochemistry (IHC) at the same laboratory, as previously described [40]. All four examined birds were males.

## Results

### Natural infection in blood collected from wild birds

Two *Plasmodium* lineages were observed in this study. Phylogenetic analysis of 21 *Plasmodium* spp. cyt *b* sequences obtained from GenBank places the two *Plasmodium* lineages used to infect and mosquitoes with in this study (HOFI_CA_JSC_1P and SPTO_CA_ELW_6P) in the clade with *P. cathemerium* (Fig. 2). This placement confirmed morphological identifications from blood smears as *P. cathemerium* belonging to the subgenus *Haemamoeba,* which is characterized by marked displacing the nucleus of the host’s red blood cell (Fig. 1) [1]. Morphologically, blood stages of the HOFI_CA_JSC_1P strain was similar to *P. cathemerium,* but differed by a 1.05 % genetic distance within the cyt *b* gene, which led us to call this isolate *P. cathemerium*-like.

The inoculation of sample SL7 BHGR into canaries 0509 and 0502 resulted in a *P. cathemerium* (lineage SPTO_CA_ELW_6P) infection, despite the fact that both microscopic and PCR screening determined that the donor was infected with *Haemoproteus* spp. and not *Plasmodium* spp. The undetected *P. cathemerium* in the original sample from the wild bird is a common phenomenon arising from light *Plasmodium*-infections that make it difficult to detect microscopically, or due to the presence of only tissue merozoites [1]. Additionally, PCR-based methods tend to bias the amplification of one parasite over another in co-infections [2, 17]. Perhaps, studies that have been conducted thus far have underestimated the actual prevalence and diversity of avian malaria parasites in avian populations. Without the development of highly sensitive species-specific primers, our understanding of the community composition of avian malaria parasites in the wild will continue to be incomplete.

### Proposed workflow of blood collection and inoculation

**Fig. 3:**
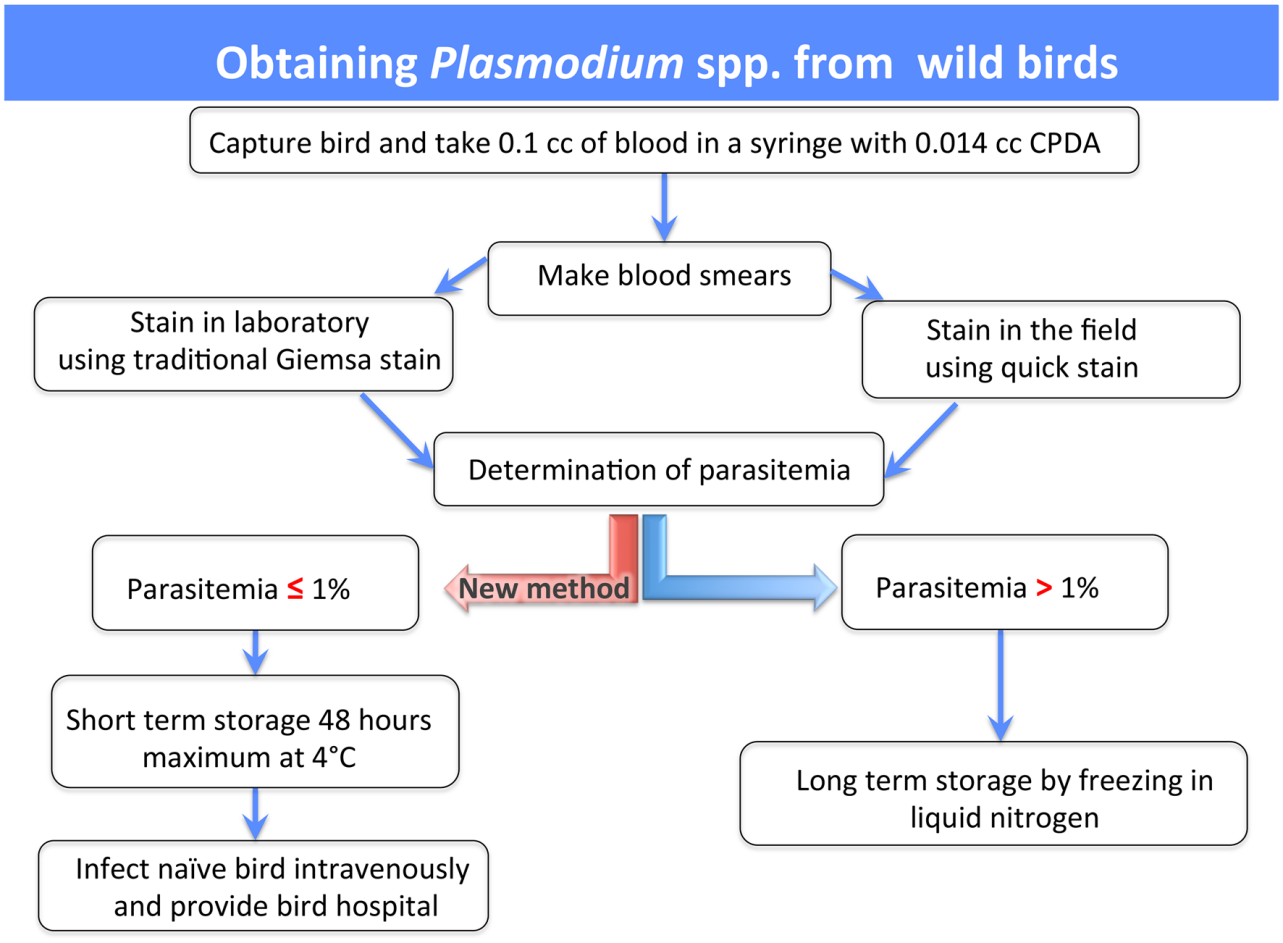
Flow chart showing the process for obtaining and preservation of *Plasmodium* spp. from wild birds. *CPDA* citrate phosphate dextrose adenine solution

After switching to the anticoagulant CPDA, there was no mortality due to complications from donor to recipient inoculations. Figure 3 provides the proposed workflow in attaining infected blood from wild birds and inoculating naïve birds. Briefly, 0.014 cc of CPDA mixed with 0.1 cc of donor blood yields viable parasites for 48 h when stored at 4 °C. Staining procedures for the blood smears will depend on whether performed in a laboratory setting or if they must be performed in the field. If performed in the laboratory, then staining methods can be followed as described by Valkiūnas et al. [19], and this staining technique will also meet the gold standards as a potential museum voucher. However, if the staining procedure must be carried out in the field, then the blood smear slides can be stained with Wright-Giemsa Stain Stat-quick (Fisher Scientific, Hampton, New Hampshire) following the manufacturer’s directions, which requires no fixation and less time for staining. This stain can help with alleviating time constraints, but it would be advisable to make extra blood smears to then stain with the method previously mentioned to meet museum voucher standards.

Once the infection status in a wild bird is established, then it is recommended to intravenously inoculate 0.04 cc of donor blood into recipient naive bird even if the parasitaemia is 0.0024 % or lower. It is advisable to provide a “bird hospital” for the recipient bird right after inoculation, for it increases the chances of survival and recovery. The bird hospital used in this study consisted of a plastic container with a circular opening on top that was covered with fine mesh to allow for airflow and prevent the bird from escaping. Two Hotsnapz (LaPorte, Indiana) were placed at the bottom of each bird hospital as a source of heat. This was especially critical to do when using donor blood that is of a different species than from the recipient. Recovery time differed considerately for each recipient but generally occurred within 10–20 min after inoculation. Inoculation of whole blood with at least 0.0024 % parasitaemia resulted in the successful establishment of an infection in the canaries.

The methods of blood preservation and inoculation into canaries within 48 h after extraction from a wild bird provides a reliable starting point that will help investigators study naturally occurring *Plasmodium* parasites without having to maintain and stress wild birds.

### Mosquito experimental infection

Canaries 0505, 0508 and 0506 were used for the experimental infections. A total of 7 *Cx. pipiens*, 6 *Cx. quinquefasciatus*, and 22 *Cx.pipiens/quinquefasciatus* hybrids fed on canaries 0505 and 0508, which were both infected with *P. cathemerium* (SPTO_CA_ELW_6P). Mosquito dissections were carried at 26 and 30 dpi. Only one dissected mosquito thorax (*Cx. pipiens/quinquefasciatus* hybrid) tested positive at 26 dpi for *P. cathemerium* that fed on canary 0505. Oocysts were not observed in the midgut and no sporozoites were visible in the salivary glands of this mosquito.

**Fig. 4:**
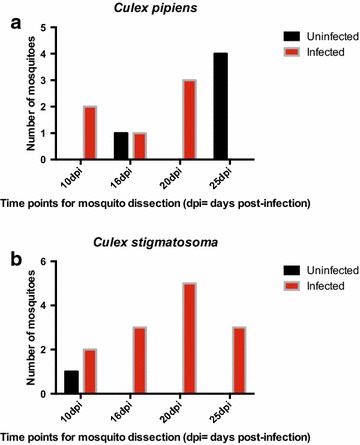
Infection status of mosquito thoraxes for (**a**) *Culex pipiens* (N = 11) and (**b**) *Culex stigmatosoma* (N = 14) that fed on canary 0506 infected with *Plasmodium cathemerium*-like lineage HOFI_CA_JSC_1P. Mosquitoes were dissected and processed at 10, 16, 20, and 25 dpi

**Fig. 5:**
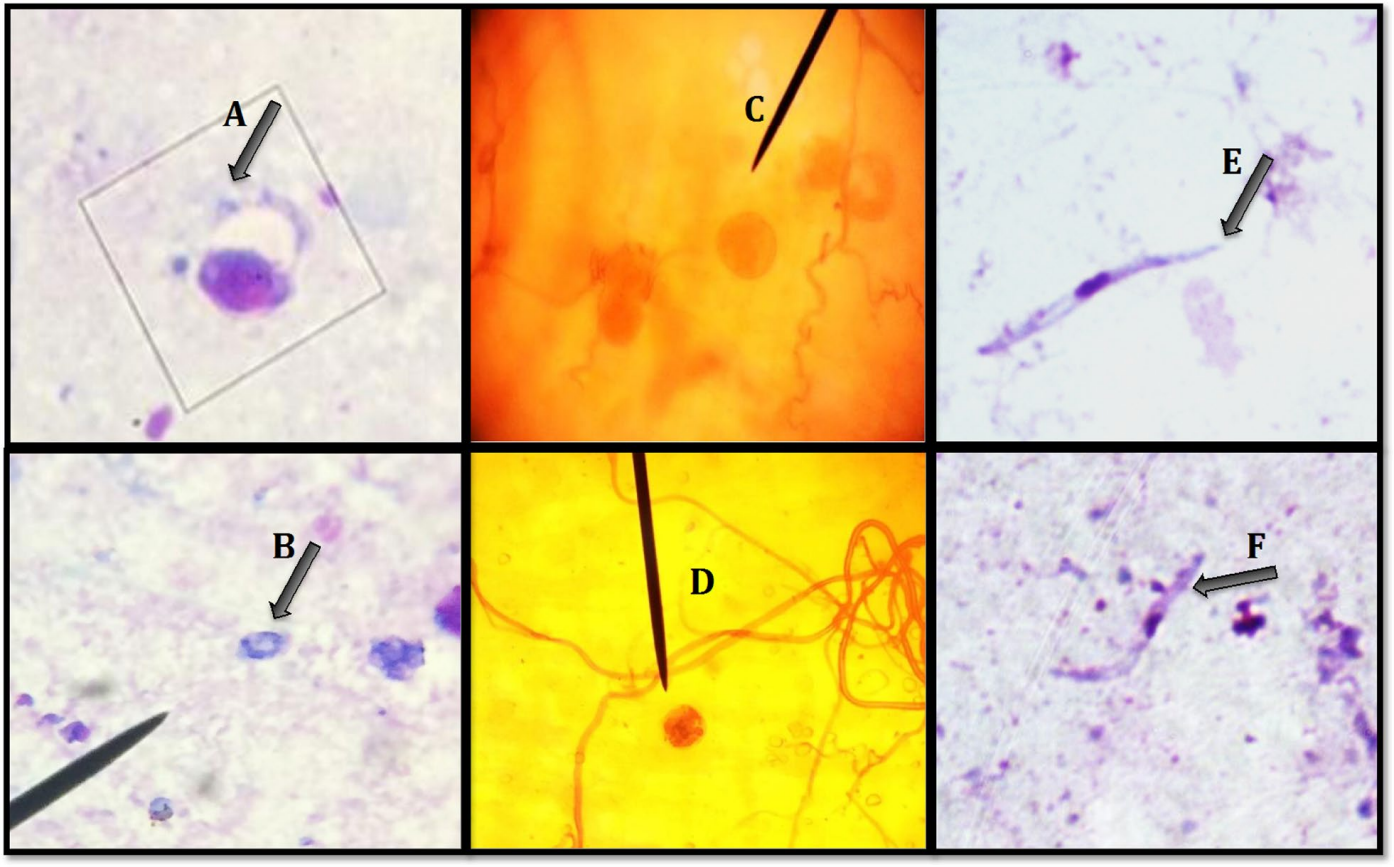
Ookinetes (*A*, *B*), oocysts (*C*, *D*), and sporozoites (*E*, *F*) of *Plasmodium cathemerium*-like (lineage HOFI_CA_JSC_1P) in *Culex pipiens* (*A*, *C*, and *E*) and *Culex stigmatosoma* (*B*, *D*, and *F*). 1000× oil immersion of Giemsa stain (*A*, *C*) and 400× of mercurochrome midgut stain (*B*)

In contrast, 13 *Cx. pipiens*, 5 *Cx. pipiens/quinquefasciatus* hybids and 26 *Cx. stigmatosoma* fed on canary 0506, which was infected with *P. cathemerium*-like lineage HOFI_CA_JSC_1P. One representative of both a *Cx. pipiens* complex and a *Cx. stigmatosoma* was sacrificed on two dpi to look for ookinetes in the midguts (Fig. 5a, b). Midgut and salivary gland dissections for the remainder of the experimentally infected mosquitoes were performed at 10, 16, 20 and 25 dpi (Fig. 4). Of the *Cx. pipiens* complex, six *Cx. pipiens* thoraxes tested positive of which three individuals had oocysts ranging from 9 to 29 in number and three were observed also to be sporozoite positive in the salivary glands. Of the 15 *Cx. stigmatosoma* tested, 12 thoraxes were parasite-positive (Fig. 4). Of these 12 thorax-positive *Cx. stigmatosoma*, six were observed to have salivary glands infected with sporozoites and the numbers of oocysts ranged from 5 to 39. Due to low numbers in this infectivity assay, the infection rate was determined by the infection status of the thorax confirmed by PCR. The infection rate of *Cx. stigmatosoma* was significantly higher than that observed in *Cx. pipiens* complex members (Fig. 4, *χ*^*2*^ = 31.9614, df = 1, *P* < 0.0001). This suggests that *Cx. stigmatosoma* is a natural vector for this lineage of *P. cathemerium*, which is the first time that it has been implicated as a competent vector for this parasite. *Cx. pipiens* is also a likely natural vector for this particular lineage of *P. cathemerium.* Figure 5 depicts ookinetes (A, B), oocysts (C, D) and salivary gland sporozoites (E, F) in *Cx. pipiens* (A, C, E) and in *Cx. stigmatosoma* (B, D, F).

Interestingly, these two small-scale infectivity assays suggest that the members of the *Cx. pipiens* complex may differ in their vector competence for these two different lineages of *P. cathemerium*. One *Cx. pipiens* hybrid was positive for *P. cathemerium* lineage SPTO_CA_ELW_6P, while six *Cx. pipiens* were positive for the *P. cathemerium*-like lineage HOFI_CA_JSC_1P. Although the sample sizes are very small and no real conclusion can be made, these data elicit the need for more experiments that will further address the observed heterogeneity in the transmission of *P. cathemerium* lineages among members of the *Cx. pipiens* complex. This is especially true when considering that Kothera et al. [41] were not able to find pure *Cx. pipiens* in California, only hybrids.

### Pathologic examination of deceased birds

#### Necropsy

The *Plasmodium* spp.—inoculated birds were in thin body condition, with minimal coelomic fat reserves, and showed moderate (canary 0509) to severe (canaries 0502 and 0505) bilateral symmetrical atrophy of the pectoral muscles. Necropsy findings in all three inoculated canaries included severe enlargement of the spleen (splenomegaly) with dark brown discoloration of the splenic parenchyma. The estimated volume of the spleen in the inoculated canaries was between 11.75 and 18.4 times greater than the volume of the organ in the control bird (Table 1). The uninfected bird was in fair body condition, with adequate coelomic and subcutaneous fat depots, and the pectoral muscles and spleen were within normal limits.

**Table 1:** Macroscopic characteristics of the spleen in three *Plasmodium* *cathemerium*–like (lineage HOFI_CA_JSC_1P) infected canaries and one, aged-matched, uninfected control

Bird ID	Infection status	Spleen			
		Shape	Length (cm)	Diameter (cm)	Approximate volume (cm^3^)
0502	Infected	Cylindrical	1.5	0.4	0.188
0509	Infected	Cylindrical	1.8	0.4	0.226
0505	Infected	Cylindrical	1.5	0.5	0.295
0507	Uninfected	Cylindrical	0.9	0.15	0.016

#### Histopathology and immunohistochemistry

**Fig. 6:**
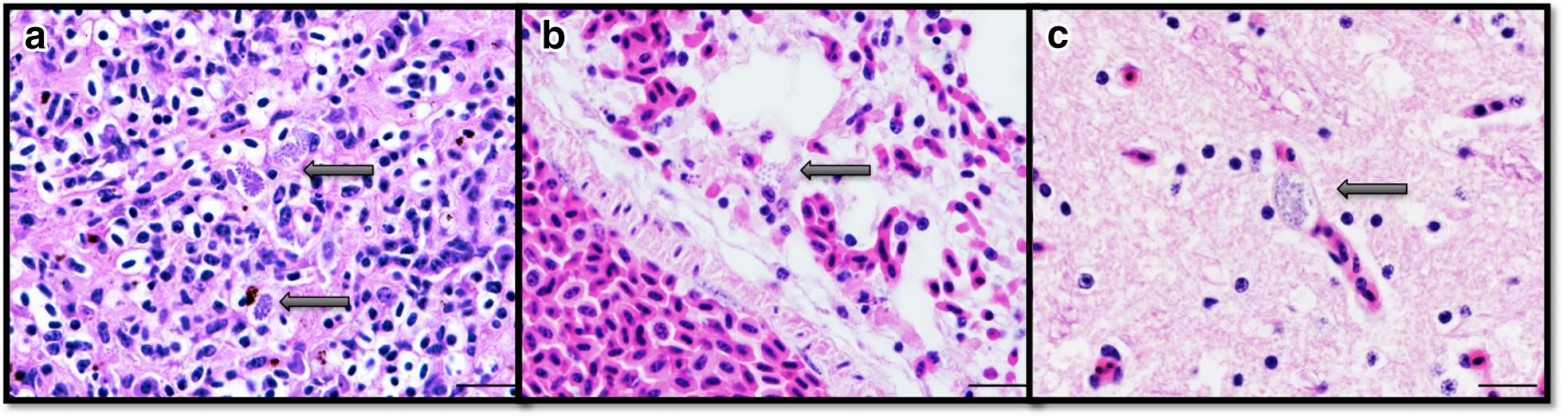
Micrographs of spleen (*A*), lung (*B*), and brain (*C*) from canary 0505 infected with *Plasmodium cathemerium* (lineage SPTO_CA_ELW_6P). Exoerythrocytic meronts (*arrows*) in histiocytes and endothelial cells (*arrows*). Haematoxylin and eosin stain, original magnification 1000× (oil). *Bars* = 10 µm

Microscopic examination in all three *Plasmodium* spp.—infected birds revealed severe lymphoplasmacytic and histiocytic splenitis and variable numbers of protozoal meronts in histiocytes and capillary endothelial cells in multiple organs, most notably, but not exclusively, the spleen, liver, lungs, and brain (Fig. 6, A–C). Exoerythrocytic meronts were round or oval, ranged from approximately 4–16 microns in diameter, often displaced the nucleus of the infected cells, and contained dozens of round to slightly elongated basophilic merozoites that ranged between 0.5 and 1 μm in diameter. Scattered splenic histiocytes contained intracytoplasmic brown granular pigment consistent with malarial pigment (haemozoin). In the two birds that had survived longer after inoculation (502 and 505) there was multifocal minimal non-suppurative encephalitis. No microscopic lesions were seen in the spleen, liver, lung or brain of the control bird. *Sarcocystis neurona*, *Sarcocystis falcatula* and *Chlamydia* spp. were not detected by IHC in any of the three birds.

## Conclusions

A key result of this study was the development of a method of collecting avian malaria parasites in the field that when preserved for 48 h post extraction from a bird still served as a viable source of infective *Plasmodium* parasites. Importantly, this infection method still proved infectious even at a very low parasitaemia (≤0.0024 %), in contrast to cryopreservation which remains infectious when parasitaemias are in excess of 1 %. This method also has the advantage of not stressing wild birds further by avoiding having to take them to the laboratory. This study provides molecular and microscopic evidence of successful development of *Plasmodium* parasites, which were preserved using this method, both in vectors and avian hosts (Figs. 5, 6).

Of the five experimentally-inoculated canaries in this study, three developed clinical signs characteristic of avian malaria and succumbed to the disease (canaries 0502, 0509, and 0505). The pathologic examinations confirmed that the *P. cathemerium* lineages developed tissue stages in multiple organs and induced typical lesions described in naturally-occurring cases of avian malaria, most notably splenomegaly and splenitis with intrahistiocytic malarial pigment [42, 43]. These results indicate that, under the reported experimental conditions, handling of the *Plasmodium* spp.—infected blood outside the avian host for up to 48 h did not seem to affect the pathogenicity of the strain. Moreover, mosquitoes were successfully infected by feeding on the canaries experimentally infected with two different lineages of *P. cathemerium*, thus confirming that the proposed method is reliable for infectivity assays. Interestingly, it was also determined that *Cx. pipiens* and *Cx. stigmatosoma* are likely natural vectors of *P. cathemerium*. *Cx. stigmatosoma* has never been identified as a competent vector for *P. cathemerium* until now. The determination was made possible by the presence of sporozoites in salivary gland preparations for these species of mosquitoes.

## Abbreviations

ATP: adenosine triphosphate
CPDA: citrate phosphate dextrose adenine solution
Cyt b: cytochrome oxidase subunit-*b*
dpi: days post infection
IACUC: Institutional Animal Care and Use Committee
PCR: polymerase chain reaction
IHC: immunohistochemistry
